# Rapid divergent evolution of internal female genitalia and the coevolution of male genital morphology revealed by micro-computed tomography

**DOI:** 10.1098/rspb.2023.2883

**Published:** 2024-01-31

**Authors:** Nadia S. Sloan, Mark S. Harvey, Joel A. Huey, Leigh W. Simmons

**Affiliations:** ^1^ Centre for Evolutionary Biology, School of Biological Sciences, University of Western Australia, Crawley 6009, Australia; ^2^ Collections and Research, Western Australian Museum, Welshpool 6106, Australia; ^3^ Biologic Environmental, East Perth 6004, Australia

**Keywords:** *Antichiropus* millipedes, evolutionary rates, genitalia, sexual selection, speciation, 3D morphometrics

## Abstract

Animal genitalia are thought to evolve rapidly and divergently in response to sexual selection. Studies of genital evolution have focused largely on male genitalia. The paucity of work on female genital morphology is probably due to problems faced in quantifying shape variation, due to their composition and accessibility. Here we use a combination of micro-computed tomography, landmark free shape quantification and phylogenetic analysis to quantify the rate of female genital shape evolution among 29 species of *Antichiropus* millipedes, and their coevolution with male genitalia. We found significant variation in female and male genital shape among species. Male genital shape showed a stronger phylogenetic signal than female genital shape, although the phylogenetic signal effect sizes did not differ significantly. Male genital shape was found to be evolving 1.2 times faster than female genital shape. Female and male genital shape exhibited strong correlated evolution, indicating that genital shape changes in one sex are associated with corresponding changes in the genital shape of the other sex. This study adds novel insight into our growing understanding of how female genitalia can evolve rapidly and divergently, and highlights the advantages of three-dimensional techniques and multivariate analyses in studies of female genital evolution.

## 1. Introduction

Male genitalia are hyperdiverse structures that facilitate the successful transfer of gametes. The rapid divergent evolution of male genitalia is thought to arise via sexual selection, with variation in the shape of male genitalia affecting their ability to engage the female in copulation, transfer sperm to the female reproductive tract and stimulate the female to use those sperm to fertilize ova [1–3]. Male genitalia are so divergent in morphology that they have long been used as a tool in taxonomy for species identification and description [4]. By contrast, female genitalia have been largely understudied [5,6]. This may be because female genitalia tend to be composed of soft, fragile tissue, and are often located internally, making them more difficult to study than the generally rigid, external structures of males [5,7]. Indeed, early work on genital evolution suggested that female genitalia are evolutionary static structures that are relatively undifferentiated between species, especially when compared to male genitalia [1].

More recently, efforts have been made to correct the male bias in studies of animal genitalia [8–10]. We now know that female genitalia can be just as divergent as male genitalia [11–15], and there is increasing evidence that female genitalia show patterns of coevolutionary divergence with male genital morphology, both among species [16–21] and within species [22–24]. While genital divergence is frequently said to be rapid, quantification of the rates of genital evolution are rare [3], and little is known of the rates of female genital divergence relative to that of males. Among pentatomid stink bugs, male genitalia appear to be diverging more rapidly than female genitalia [25]. By contrast, a study of thynnine wasps found that female and male genitalia are diverging at equivalent rates [26], whereas a study of onthophagine dung beetles found that female genitalia can evolve significantly faster than male genitalia [27]. Across beetles generally female genital shape appears to drive the evolution of male genital morphology [19]. Although these studies point to an important role for female genitalia in the rapid and divergent evolution of male genitalia, more studies of the rates of female genital evolution relative to that of males will be required before any general patterns can be uncovered.

Recent advances in micro-computed tomography (micro-CT) and three-dimensional (3D) shape analysis are offering new opportunities for the quantification and analysis of female genitalia and their interactions with male genital traits [28–31]. In the millipede *Antichiropus variabilis*, micro-CT and 3D shape analyses have revealed that among seven populations across the species range, both female and male genitalia are more divergent in shape than would be expected from neutral genetic divergence, consistent with a pattern of directional selection acting on both female and male genital shape [32]. While there was evidence of correlated divergence between female and male genitalia, male genital divergence tended to be greater than female genital divergence suggesting that male genital morphology may be diverging more rapidly than female genital shape. Here we build upon this previous work by examining female genital evolution in *Antichiropus* millipedes on a macroevolutionary scale. We use micro-CT scans of female and male genitalia and a landmark free methodology [33] to quantify variation in genital shape among 29 species of *Antichiropus*. We construct a molecular phylogeny of the genus and use this phylogeny to compare the rates of evolutionary divergence in female and male genital shape, and their coevolutionary divergence. In so doing, we provide a novel contribution to the growing body of work that is revealing the dynamic nature of female genital evolution.

## 2. Material and methods

### (a) *Antichiropus* millipedes

The genus *Antichiropus* is a highly speciose genus of paradoxosomatid millipede endemic to Western Australia. Currently, 72 species have been formally described and there are at least a further 120 species catalogued in the collections of the Western Australian Museum [34–36]. Most species are short-range endemics with very low vagility and little sympatry [37]. During mating interactions, the male charges his secondary genitalia, a pair of modified legs known as the gonopods, with ejaculate from his primary genitalia and then inserts the gonopods into gonopores on the third body segment of the female (electronic supplementary material, figure S1). The tips of the gonopods interact with internal sperm storage structures, the receptacula, inside the female where sperm are stored prior to their use in fertilization [28,32]. We quantified shape variation in the gonopods and the receptacula of 29 species of *Antichiropus*.

### (b) Sample acquisition and preparation

We sampled species of *Antichiropus* from the southwest of Western Australia, broadly from an area delimited by a boundary from Eneabba, 274 km north of Perth, and Caiguna, 1090 km southeast of Perth. This southwest bioregion includes the majority of known species in the genus, with the northern Pilbara bioregion having an additional 31 described species [36]. We included all species from the southwestern region for which both male and female specimens were available. Specimens were sourced either from the Western Australian Museum or from extensive field collections conducted in southwest Western Australia, between April and September of 2017, 2018 and 2019 (electronic supplementary material, table S2, figure S2). All specimens were assigned to species based on the gonopod morphology of males (C. Car, Western Australian Museum). Our sample consisted of both described species [34,35], and species that are recognized by the Western Australian Museum but are yet to be formally described. In total, we had material from 35 species (approx. 20% of the known taxonomic diversity) with which to construct a phylogeny, 29 of which we had samples of both males and females with which to quantify genital shape. All museum specimens were stored in 75% ethanol. Freshly collected male specimens were stored in 100% ethanol. The head and legs from freshly collected female specimens were dissected and stored in 100% ethanol and the body was preserved in 4% paraformaldehyde (PFA).

We made three-dimensional (3D) micro-computed tomography (micro-CT) scans of the female genitalia (*n*_total_ = 131, mean = 4.5, range = 1–12) and male genitalia (*n*_total_ = 136, mean = 4.7, range = 1–12) of *Antichiropus* millipedes from 29 species (see electronic supplementary material, table S1). Prior to micro-CT imaging, collum area was acquired by taking photographs of individuals (females: *n*_total_ = 126, mean = 4.3, range = 1–11; males: *n*_total_ = 133, mean = 4.6, range = 1–12) in a lateral view using a binocular microscope, and using the area measurement tool in Leica Application Suite v4.11.0 (Leica Microsystems, Switzerland) (electronic supplementary material, figure S3). Not every individual was able to have collum area measured, as some specimens were damaged. As such, the sample sizes available for each species, sex and trait varied (electronic supplementary material, table S1).

For female millipedes, body rings 1–4 (containing the genitalia) were removed, and for male millipedes, the left gonopod was removed. Female segments that had been preserved in 4% paraformaldehyde (PFA) were individually immersed in a 3% solution of Lugol's iodine (I_2_KI); 1% weight by volume (w/v) I_2_ and 2% w/v KI in dH_2_O. Female segments that had been preserved in ethanol (museum specimens), as well as all male gonopods, were immersed individually in 1% w/v I_2_ in 100% ethanol (I2E). Every sample was immersed in 4 ml of their respective stains in a 6.5 ml screw cap, plastic test tube. All tubes were wrapped in aluminium foil as both Lugol's iodine and I2E are photosensitive, and then samples were placed on a plate stirrer at room temperature for 24 h prior to imaging.

### (c) 3D-imaging

X-ray micro-computed tomography was conducted using a Zeiss Versa XRM-520 X-ray microscope (Zeiss, Oberkochen, Germany) at the Centre for Microscopy, Characterization and Analysis at the University of Western Australia. Full details of sample mounting and scanning parameters can be found in the electronic supplementary material. The software XMReconstructor (Zeiss) was used to construct the 3D volumetric data upon completion of scanning. A beam hardening constant of 0 and automatic center shift were used.

The manual segmentation of female and male genitalia was conducted using Avizo v9.2.0 software (Thermo-Fisher Scientific, USA), where the right female receptaculum and left male gonopod were isolated from each 3D volume. Full details of the workflows can be found in the electronic supplementary material. Once segmentations were complete, each female receptaculum and male gonopod was exported as a surface mesh file in the Polygon File format (.ply). Each .ply file was then checked for holes and non-manifold edges in MeshLab (v2021.10) [38] so that no surfaces with missing data were included in downstream analyses.

### (d) 3D-geometric Morphometric analysis

The GPSA software (version 20160308) [33] was used to perform both a generalized Procrustes surface analysis (GPSA) and a Gower's principal coordinates analysis (PCOORD) on all extracted surfaces. The GPSA software is designed to analyse whole surface scans, as opposed to more traditional landmark based data [33]. The software can also produce average surface heatmaps for female and male genitalia. While a relatively new approach to 3D shape analysis, GPSA is proving to be a highly effective approach for the comparison of complex structures that have few of the homologous structures necessary for traditional landmark based approaches [39–41], and it has recently been used in the analysis of intraspecific genital shape variation in *A. variabilis* [32]. The GPSA produced 130 axes of variation for females and 135 axes for males. Using the scores on these axes of variation, we conducted multivariate analyses of variance (MANOVA) for both female and male genital measures to determine whether species differed significantly in trait shape. The MANOVAs were executed via permutation using RRPP (version 1.0.0) [42,43] and listing ‘species' as the independent variable.

### (e) Genetic analyses and phylogenetic reconstruction

*Antichiropus* species typically have limited vagility and are often short-range endemic species [37], so that females collected at the same locality as males can be assigned to species with high confidence based on the taxonomic assessment of males. However, to add confidence to the taxonomic assignment of females, we extracted and sequenced DNA from all females of 17 species whose identities had not previously been confirmed using sequence data (electronic supplementary material, table S6). DNA was extracted from dissected legs (one to five legs) and heads using the DNeasy Blood and Tissue kit (Qiagen, Chadstone, Australia) following the manufacturer's instructions. A Qubit 4 Fluorometer (Invitrogen, Singapore) was subsequently used to examine DNA quality and quantity from all specimens. All specimens were sequenced for the mitochondrial DNA gene, COI. Polymerase Chain Reaction (PCR) was used to amplify the COI gene, using two different primer pairs depending on the sample (see electronic supplementary material, table S6). The primer pair LCOI490 (5′-GGTCAACAAATCATAAAGATATTGG-3′) and HCO2198 (5′-CTAAACTTCAGGGTGACCAAAAAATCA-3′) [44] was used for 48 samples, and NemF1 and NemR1 (unpublished, Helix Molecular Solutions Pty Ltd, Perth, Australia) for a further 17 samples. Amplification of the COI gene using the LCOI490 and HCO2198 primers was conducted in 25 µl reactions containing 0.25 µM of each forward and reverse primer, 0.2 mM dNTPs, 0.75 mM MgCl_2_, 1 × PCR buffer, 0.4 mg ml^−1^ BSA (Fisher Biotec, Wembley, Australia), 0.05 U Platinum Taq DNA Polymerase (Thermo Fisher Scientific, USA) and 5 µl of template DNA. Alternative amplification of the COI gene using the NemF1 and NemR1 primers was conducted in the same manner as above but using 1 mM MgCl_2_. PCR reactions for LCOI490 and HCO2198 primers ran at 94°C for 3 min, then 37 cycles at 94°C for 45 s; 37 cycles at 48°C for 45 s; 37 cycles at 72°C for 45 s, and then finished at 72°C for 5 min. PCR reactions for NemF1 and NemR1 primers ran at 94°C for 2 min, then 40 cycles at 94°C for 30 s; 40 cycles at 56°C for 20 s; 40 cycles at 72°C for 30 s; and then finished at 72°C for 2 min. PCR products were visualized on 2% agarose gels buffered in TBE. Dual direction Sanger sequencing was carried out at the Australian Genome Research Facility (AGRF Ltd, Perth, Australia). Sequences were edited, and pairwise DNA alignments were performed using Geneious Prime v2020.2.4 (Biomatters, Auckland, New Zealand). The mean similarity between females and the males with which they were collected was 98.3% (range 100–94.5) giving us confidence in their taxonomic assignment (electronic supplementary material, table S6).

Currently, there is no published phylogeny for *Antichiropus*. Our samples came from a broad geographical range and included both described and undescribed species. The final sample available for our prospective phylogenetic reconstruction consisted of 35 *Antichiropus* species, approximately 20% of known taxonomic diversity. For all recently caught specimens, DNA was extracted as described above. For specimens whose species identities were unknown (both recently caught and museum supplied; see electronic supplementary material, table S2), we obtained sequence data from four mitochondrial genes (cytochrome c oxidase subunit 1 [COI], cytochrome c oxidase subunit 3 [COIII], cytochrome B [CytB] and 12S rRNA [12S]), and one nuclear gene (28S rRNA [28S]) for use in phylogenetic reconstruction, following the protocols outlined by Car *et al*. [36] (see electronic supplementary material, table S5 for Genbank accession numbers).

Alignments were carried out using the MAFFT plug-in in Geneious [45]. As the ribosomal genes 12S and 28S had regions that were difficult to align, the program G Blocks [46,47] was used to remove phylogenetically uninformative sites for all rRNA alignments. The web tool was chosen for this task (http://molevol. cmima.csica.es/castresana/Gblocks_server.html), and the settings allowed for gaps and larger blocks. The resulting alignments were: 12S (326 bp); 28S (339 bp); COI (552 bp); COIII (628 bp) and CytB (656 bp).

Phylogenetic analyses require a phylogeny that has been calibrated using a molecular clock to provide temporal context. Typically, two options are available for calibrating phylogenies; using a set of known fossils, or using molecular rates from similar taxa. Choosing a calibrating method upon which to build our phylogeny was difficult, given there are no known estimates of millipede divergence rates in the literature, and there are no fossil records with which to date the phylogeny. In addition, estimates for divergence rates for invertebrates are numerous and variable [48–52]. Therefore, we conducted our analyses using two commonly cited invertebrate divergence rates, 1.4% per million years (%/Ma) [49] and 2.3%/Ma [53]. These rates were used in two separate molecular clock analyses in BEAST2 v2.4.3 [54]. BEAST 2 xml files were constructed in BEAUti 2 after extensive preliminary runs and testing of convergence using Tracer [55].

The data were partitioned based on gene and codon position, and the BEAST Model Test method was assigned to select the most appropriate site model. A relaxed lognormal clock was used, with a normally distributed ucldMean value of 0.007, and a *σ* of 0.001, corresponding to a 95% CI range of 0.0054 to 0.0086. The ucldStdev prior was set to an exponential distribution and mean of 2.0. The Birth Death tree model was used, and the analysis was run for 50 million chains. After testing for convergence using Tracer, a Maximum Clade Credibility tree was constructed with Tree Annotator, with a burn in of 10%, PP limit of 0.7, and median node heights. The final tree was viewed and edited in Figtree [56]. Repeated runs recovered similar topologies and times for supported nodes. The trees were rooted on *Akamptogonus novarae*. The final tree is provided in electronic supplementary material, figure S6 of the online supporting material. Note that of the 35 species in the phylogeny, both female and male genital shape data were available for 29 species. Prior to phylogenetic analyses, the tree was pruned in R using the package ‘ape' and the ‘drop.tip' function.

### (f) Phylogenetic analyses

All phylogenetic analyses were conducted using the species mean scores on the axes of variation in male and female genital shape, and male and female collum areas. The number of axes from the GPSA for male genitalia was reduced to the same number of axes as females (we used only the first 130 of the 135 axes) in order to create a symmetrical matrix for all phylogenetic analyses.

We assessed phylogenetic signal to determine if more closely related species shared similar genital shapes and collum areas. The genital shape data in this analysis was highly multivariate, so we used Adams' *K*_mult_ statistic [57] to assess phylogenetic signal. The *K*_mult_ statistic is a generalization of Blomberg's *K* statistic [58] and is more suited to highly multivariate data. If *K*_mult_ = 0, there is no phylogenetic signal in the data; if *K*_mult_ = 1, traits are evolving according to the phylogeny as expected under Brownian motion [57]. For the collum area data, as these data were univariate, Blomberg's *K* statistic was returned [58]. We used the function ‘physignal' in the R package geomorph (version 4.0.6) [59,60], using 1000 iterations. Significant values indicate that traits are evolving significantly differently from *K*_mult_ = 0. We also calculated the phylogenetic signal effect size, *Z*, using the function physignal.z with *λ* = 1 (which provides an effect size for the value of *K* estimated by the function physignal), and compared the phylogenetic signal effect sizes for male and female genitalia and for male and female collum area using the function compare.physignal.z.

We compared the rates of evolution between female and male genitalia, as well as between female and male collum area using the ‘compare.multi.evol.rates' function in geomorph [59,60] and 1000 iterations. This analysis produces R, the evolutionary rate ratio as well as *σ*^2^, the multivariate rate of change for each trait. We set the argument ‘Subset = False' to specify that traits came from different subsets. In each analysis, we concatenated the 2D matrices of female and male genital shape data, as well as female and male collum data. Following the estimations of evolutionary rates between traits, we also calculated 95% confidence intervals around *σ*^2^ values for each trait by bootstrapping the data (using 1000 iterations) that was used to calculate the species means.

We tested for correlated divergence in female and male genital shape as well as female and male collum area across species. To do so, we conducted phylogenetic two-block partial least-squares analyses (pPLS) using the function ‘phylo.integration’ in the package geomorph [59,60], and 10 000 iterations.

## 3. Results

### (a) Genital shape

**Figure 1.**
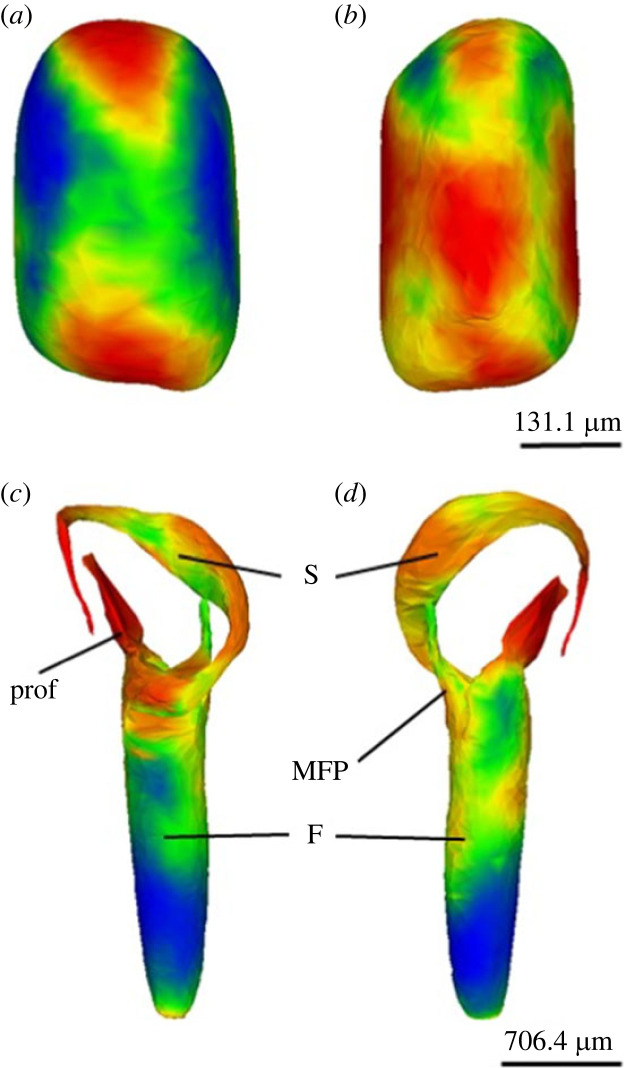
Average surface heatmaps produced by the GPSA. Red vertices reflect areas of high variation, whereas blue vertices reflect areas of low variation. (*a*) Female receptaculum anterior view. (*b*) Female receptaculum posterior view. (*c*) Male gonopod posterior view; prof, prolongation of the femur; F, femur; S, solenomere. (*d*) Male gonopod lateral view; MFP, main femoral process.

The GPSA returned 130 axes of variation for females and 135 axes of variation for males (electronic supplementary material, figure S7). The GPSA produced heatmaps showing the average surface shape for both female and male genital shape (figure 1). For females, the greatest shape variation occurred in the ridges at the top and bottom of the receptaculum and at the posterior of the receptaculum (figure 1*a,b*). For males, the gonopod showed the greatest shape variation in the tip of the solenomere (S) and in the prolongation of the femur (prof) (figure 1*c,d*). The femur (F) showed the least amount of shape variation (figure 1*c,d*).

**Figure 2.**
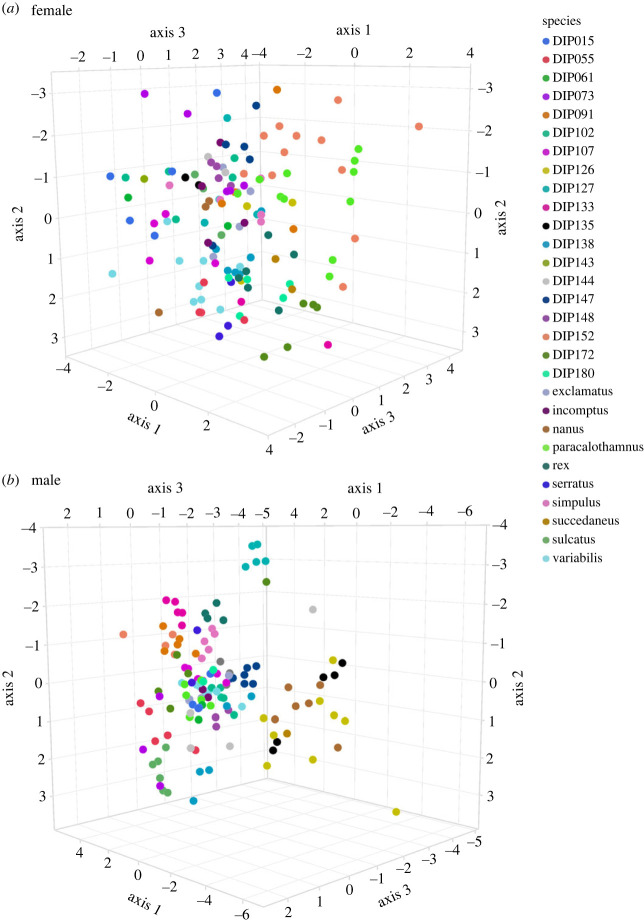
Scatterplots of the first three principal axes from GPSA showing species separation (*a*) for females, where the first three axes explained 24.7% of the variation in genital shape, and (*b*) for males, where the first three axes explained 28.7% of the variation in genital shape.

The axes of shape variation produced by the GPSA were used in permutation MANOVAs to test statistically for differences among species in female and male genital shape. Using 1001 permutations, both receptaculum shape (Pillai = 9.03, *Z* = 14.76, *p* < 0.001) and gonopod shape (Pillai = 11.83, *Z* = 20.19, d.f. 28, *p* < 0.001) were found to vary significantly among species of *Antichiropus*. Visual inspection of the first three axes show that the separation among species was clearer for gonopod shape than it was for receptaculum shape (figure 2).

### (b) Collum area

While there was significant variation among species in body size, measured as collum area, there was no evidence of sexual dimorphism in body size (ANOVA: species, *F*_28,229_ = 49.68, *p* < 0.001; sex, *F*_1,229_ = 0.853, *p* = 0.357).

### (c) Phylogenetic signal

**Figure 3.**
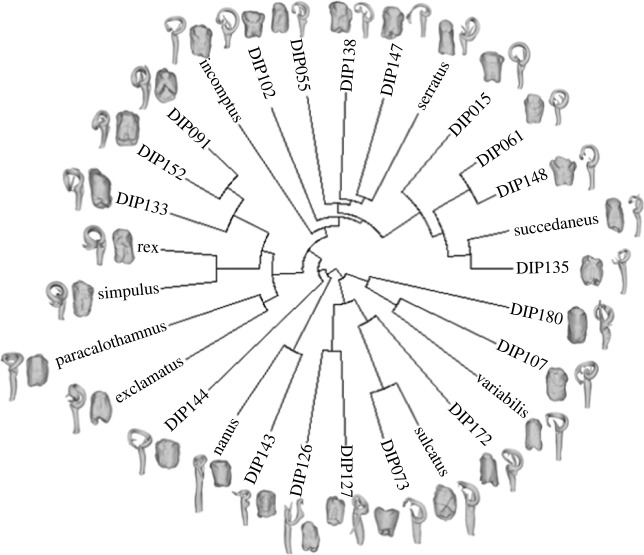
Morphological variation in female receptaculum and male gonopod shape seen across the pruned phylogenetic tree of *Antichiropus* millipedes. The full phylogeny, including posterior support for branch values, can be found in electronic supplementary material, figure S6.

Male genital shape had a significant phylogenetic signal (*K*_mult_ = 0.72, *p* = 0.001, effect size *Z* = −0.69) indicating that in more closely related species, males shared more similar genital shape (figure 3). Consistent with the weaker separation of species observed in figure 2*a*, female genital shape had a lower phylogenetic signal that lacked statistical significance (*K*_mult_ = 0.58, *p* = 0.368, effect size *Z* = 0.97). Nevertheless, there was no significant difference in the phylogenetic signal effect sizes between males and females (*P* = 0.482). The phylogenetic signals for collum area were not statistically significant for either females (*K* = 0.53, *p* = 0.710, effect size *Z* = −0.51) or males (*K* = 0.70, *p* = 0.188, effect size *Z* = 0.88), and the phylogenetic signal effect sizes for collum area did not differ significantly between the sexes (*p* = 0.328).

### (d) Rates of trait evolution

Analyses using divergence rates of 1.4% and 2.3% returned qualitatively similar conclusions, and so the results presented here are based on a divergence rate of 1.4% Ma^−1^ (see electronic supplementary material, table S7 for results based on 2.3% Ma^−1^). A significant difference in evolutionary rate was found between female and male genitalia (evolutionary rate ratio, *R* = 1.15, *p* = 0.001). Evolutionary rate comparisons revealed that male genitalia are evolving faster than female genitalia (table 1). These evolutionary rate parameters indicate that male genitalia have evolved 1.2 times faster than female genitalia. By contrast, there was no significant difference in evolutionary rate detected between female collum area and male collum area (evolutionary rate ratio, *R* = 1.20, *p* = 0.566; table 1).

**Table 1.** Evolutionary rates of change for female genital shape, male genital shape, female collum area and male collum area across 29 *Antichiropus* species. Post hoc pairwise comparisons showed a significant difference in evolutionary rates between female and male genital shape only.

	evolutionary rate parameter (*σ*^2^)	s.d.
female genital shape	0.470	0.032
male genital shape	0.541	0.031
female collum area	0.006	0.001
male collum area	0.007	0.001

### (e) Correlated trait evolution

**Figure 4.**
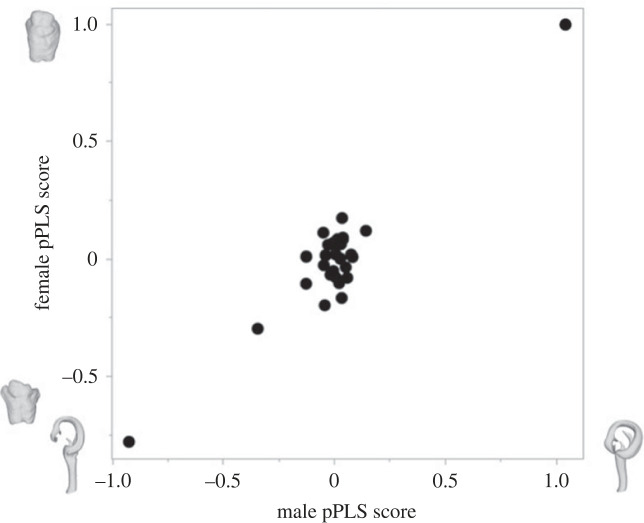
Plot returned from the phylogenetic two-block partial least-squares analyses showing the correlated evolution between female and male genital shape across 29 species of *Antichiropus*. The gonopod and receptaculum for species (DIP061 and DIP148) at the extremes of the shape variation are shown.

**Figure 5.**
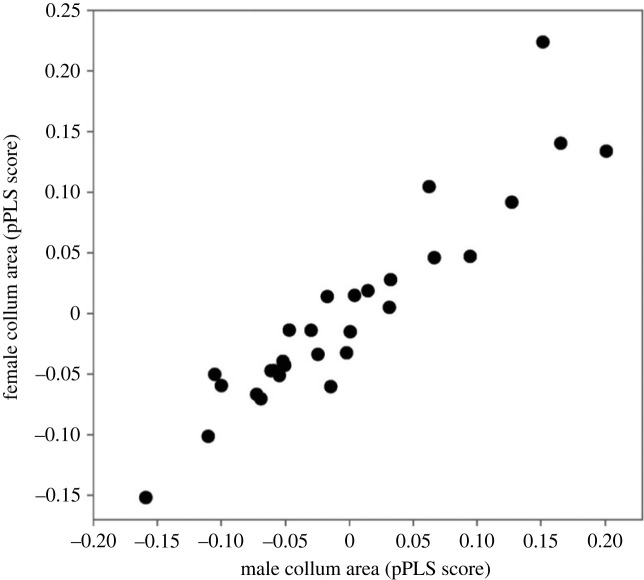
Plot returned from the phylogenetic two-block partial least-squares analyses showing the correlated evolution between female and male collum area across 29 species of *Antichiropus*.

Divergence in female and male genital shape was strongly correlated across the phylogeny (phylogenetic two-block partial least-squares (pPLS) analyses, pPLS_corr_ = 0.94, *p* = 0.002; figure 4). This suggests that changes in genital shape in one sex are associated with corresponding changes in the genital shape of the other sex. Two species (DIP061 and DIP148) show much greater coevolutionary divergence than all other species (figure 4). Repeating the analysis with these two species removed returned similar results to the full dataset (pPLS_corr_ = 0.92, *p* = 0.001). Divergence in female and male collum areas was also highly correlated across the phylogeny (pPLS_corr_ = 0.93, *p* < 0.001; figure 5).

### (f) Sensitivity analysis

Our sample sizes for each species and trait varied widely due to the availability of specimens (electronic supplementary material, table S1). To determine whether our conclusions might be affected by sampling bias, we standardized our sampling to roughly 4 individuals per species and trait and re-ran all the above analyses. The results did not differ quantitatively from those reported for the full dataset (see analyses in the electronic supplementary material).

## 4. Discussion

We used micro-computed tomography (micro-CT) and molecular phylogenetic reconstruction to analyse divergence in the shape of genitalia in female *Antichiropus* millipedes, and contrasted the rate of female genital divergence with that of males. For both females and males we found rapid evolutionary divergence in genital trait shape. Male gonopod shape had a relatively stronger phylogenetic signal than receptaculum shape, and appears to be evolving 1.2 times faster than receptaculum shape. In *Antichiropus*, the receptaculum of the female and gonopod of the male interact during copulation [28], and we found evidence of significant correlated evolution in the shape of these female and male genital traits across species.

Studies of genital evolution have traditionally focused on male genital traits [5]. It is well established that male genitalia are highly divergent both within [24,61–65] and among species [66–71]. Using advances in micro-CT scanning technology to quantify the shape of an internal female genital trait, we have shown that there has been a rapid and divergent evolution of female genital morphology among 29 species of *Antichiropus* millipedes. Our findings add to a growing body of work that is revealing how female genitalia exhibit patterns of rapid and divergent evolution similar to those seen in males.

We found that divergence in the shape of the female receptaculum was significantly correlated with divergence in the shape of the male gonopod, indicating that these two traits are coevolving across *Antichiropus* species. The GPSA produced average surface heatmaps of female and male genitalia that revealed areas where the most shape variation occurred. On the female receptaculum, most of the variation was found on the ridges at the dorsal and ventral regions of the structure. For the male gonopod, the tip of the solenomere and the prolongation of the femur showed the greatest amount of shape variation. Previous work on *Antichiropus variabilis* visualized the copulatory fit between female and male genitalia and found close morphological correspondence between the tip of the solenomere and the receptaculum [28]. Furthermore, we have shown that the same aspects of shape variation are diverging more rapidly than would be expected from neutral divergence across seven populations of *A. variabilis*, and that population divergence in female and male genital shape are significantly correlated [32]. Our phylogenetic analysis shows how the microevolutionary divergence across populations of *A. variabilis* is reflected in the macroevolutionary divergence occurring across species in the genus. Collectively, the results support the suggestion that correlated divergence in genital traits among isolated populations of *Antichiropus* millipedes may be contributing to speciation in this exceptionally speciose genus of short-range endemic millepedes [72].

While many studies have examined divergence in genital shape, only a small number of studies have actually quantified the rates of genital divergence [25,27,67,68]. Fewer still have estimated the rates of female genital evolution [25–27]. Our data revealed a significant difference in the rate of evolution between female and male genitalia across *Antichiropus*, with the shape of the male gonopod diverging around 1.2 times faster than the shape of the female receptaculum. These results are an important addition to a small group of studies where rates of female and male genital evolution appear to be highly variable. Consistent with our findings for *Antichiropus* millipedes, the male genitalia of pentatomid stink bugs are evolving faster than female genitalia [25]. However, the reverse has been reported for onthophagine dung beetles [27] and no difference in rates of genital divergence was found in a study of thynnine wasps [26]. The variability in these findings and the limited number of studies reporting rates of genital evolution highlights strongly the need for more female focused genital evolution studies that address the coevolution of female and male genital structures.

Genital evolution is currently thought to occur mainly via sexual selection [1–3,73]. Consistent with directional selection acting on the receptaculum and gonopod of *Antichiropus* species, we previously showed that in *A. variabilis*, female and male genitalia appear to be evolving more rapidly than would be expected under neutral genetic drift [32]. Moreover, the male gonopod was found to be more divergent among populations than the female receptaculum [32]. The greater rate of macroevolutionary divergence in the male gonopod across the *Antichiropus* phylogeny supports the idea that male genitalia may be under stronger selection than female genitalia. What is currently less clear, is the exact mechanism(s) of selection responsible for driving genital evolution among species of this genus. Possible mechanisms of sexual selection include cryptic female choice, sperm competition and sexual conflict, and these mechanisms need not be mutually exclusive [2,3,8]. Cryptic female choice occurs when females bias the paternity of their offspring towards preferred males during and after copulation [74]. Sperm competition occurs when the sperm from multiple males compete for access to the ova of a female [75,76], and sexual conflict can arise when the interests of females and males differ over who should fertilize ova [77,78]. Previous work on *A. variabilis* found that variation in paternity success depended upon male genital shape [79]. Moreover, among population variation in gonopod shape affected both a male's ability to engage the female in copulation and competitive fertilization success [72]. The divergent and correlated evolution of female and male genitalia both among populations and species of *Antichiropus* is thereby likely to involve one or more of cryptic female choice, sperm competition and sexual conflict. A correspondence between male and female genital shape is also predicted from the lock-and-key hypothesis, where female genitalia act as a species isolating mechanism and stabilizing selection maintains male and female genital fit [1]. Lock-and-key evolution seems less likely to explain the patterns of divergence reported here, given our within species study of *A. variabilis*; among populations of *A. variabilis*, phenotypic divergence (*P*_ST_) was found to be greater than neutral genetic divergence (*F*_ST_) for both females and males, indicating that genitalia are evolving under directional selection [32]. If lock-and-key evolution were occurring, *P*_ST_ is expected to be less than *F*_ST_, indicative of stabilizing selection for the species average shaped genitalia [1].

Millipedes are polygynandrous [80] which creates an environment within which post-mating sexual selection can occur. *Antichiropus* may use their gonopods to remove or displace the sperm of rival males, which would be consistent with the last male paternity bias in *A. variabilis* [79]. Sperm removal structures have been documented previously in the males of insects [81–84], and have also been found in one species of millipede [85]. In calopterygid damselflies, the mechanical stimulation of internal female genital structures is required for the release of rival sperm from the female's sperm stores before a male can remove it from the female's reproductive tract [86]. If *Antichiropus* females also assessed stimulation delivered by male gonopods and biased paternity towards males whose gonopods achieved a better ‘fit', paternity success would depend on gonopod shape, as found in *A. variabilis* [79]. Under this scenario, the receptaculum could be seen as a female choice trait favouring the evolution of the male gonopod via sexual selection. Alternatively, the female receptaculum shape may be under directional natural selection, for example if receptaculum shape contributed to the ability of females to effectively store and fertilize their ova. Changes in receptaculum shape driven by natural selection on females for fertilization efficiency would in turn impose sexual selection on male gonopods, if changes in receptaculum shape affected male ability to gain paternity. Rapid evolution of male genital morphology in response to naturally selected changes in female genital shape has been found in *Drosophila suzukii* [87] and many beetles [19], and would be consistent with the greater rates of genital shape evolution in male *Antichiropus* relative to females.

We also found a pattern of correlated evolution in body size, measured as collum area, among *Antichiropus* millipedes. We measured collum area as a trait more likely to be under natural selection. Traits under sexual selection have been shown to have faster rates of divergence than traits under natural selection [27,88] and so provide a useful comparator for testing the hypothesis that animal genitalia are subject to especially rapid evolutionary divergence. Indeed, both female and male genitalia evolved faster than female and male collum areas across the *Antichiropus* phylogeny. Nevertheless, there are two caveats that must be considered with these comparisons. First, body size could also be under sexual selection to some extent. For example, male millipedes may be subject to sexual selection during pre-mating competitive interactions [89], which can lead to the evolution of male biased sexual size dimorphism [90]. However, we found no evidence for a difference in collum area between females and males. Moreover, we found that the rate of divergence in body size did not differ between the sexes, and that female and male body size exhibited a strong pattern of correlated evolution across the genus. A second caveat to the contrast between rates of evolution in collum area and genital shape is that genital traits were quantified in three-dimensions and so were multivariate traits, while collum area was a univariate two-dimensional trait. Therefore, any comparisons drawn between genitalia and collum area remain tentative and future work should aim to incorporate three-dimensional measurements of a naturally selected trait.

In conclusion, we found that for *Antichiropus* millipedes, both female and male genitalia are evolving rapidly and divergently across the genus. We also found that although male and female genitalia exhibit correlated evolution, male genitalia are evolving significantly faster than female genitalia, as might be expected if male genitalia are subject to strong directional sexual selection. Our study highlights the utility of micro-CT scanning technology to quantify otherwise inaccessible female genital structures, and provides rare insight into the patterns of female genital evolution and their coevolution with male genitalia. Correcting the sex bias in the study of genital evolution is important for a comprehensive understanding of the roles of natural and sexual selection in the diversification of genital traits and their fundamental role in speciation.

## Data Availability

Data are available at the Dryad Digital Repository: (https://doi.org/10.5061/dryad.hx3ffbgkq) [91]. Additional methods and analyses are provided in electronic supplementary material [92].
